# Administration of Systemic Antibiotics for Dental Treatment in Kosovo Major Dental Clinics: A National Survey

**DOI:** 10.1055/s-0041-1735931

**Published:** 2022-01-11

**Authors:** Lirim Mustafa, Hilmi Islami, Ivana Sutej

**Affiliations:** 1 Department of Health Management – Economy, School of Dental Medicine, University of Zagreb, Zagreb, Croatia; 2 Pharmacology Department, Medical Faculty, University “Hasan Prishtina”, Kosovo, Republic of Kosovo; 3 Department of Pharmacology, School of Dental Medicine, University in Zagreb, Croatia

**Keywords:** antibiotics, dentistry, resistance, prescriptions

## Abstract

**Objective** Antibiotics misuse and a high level of antibiotics resistance is observed worldwide, but particularly in developing countries. Kosovo in the last decade is facing challenges regarding antimicrobial resistance. The purpose of the present study was to investigate patterns of antibiotics prescriptions of dentists in Kosovo's major dental clinics.

**Materials and Methods** For Kosovo's prescribing pattern, data collection was obtained from 10 Regional Dental Clinics and a Tertiary Health Center regarding patients who were prescribed antibiotics in the years 2015 to 2019. Data analysis was performed by using descriptive statistics and was processed by using MS Excel.

**Results** Most prescribed antibiotic during the observed period from 2015 to 2019 in Kosovo was amoxicillin, although a drastic increase of amoxicillin with clavulanic acid—as a broad-spectrum antibiotic—is observed. The trend of antibiotics use in tertiary health institutions is in an overall decrease in Kosovo with an exception in the year 2017. Despite this overall decrease, inconsistency in prescribing is observed when the pattern is analyzed for each region separately. The highest number of patients in health care dental clinics received antibiotics for maxilla-related health conditions and the lowest number of them for oncologic ones.

**Conclusion** The patterns of antibiotics prescriptions by dental practitioners in Kosovo during the years 2015 to 2019 are fluctuating. Compared with the global health care standards, the irrational use of antibiotics in dental health care clinics in Kosovo still exist and this issue should be further addressed by respective actors.

## Introduction

Antibiotics are a precious medicine for the treatment of spreading bacterial dental infections. Nowadays, the appropriate prescription of antibiotics has become a challenge in most health care systems because different studies have reported that antibiotics are often irrationally overprescribed, which form the basis of antimicrobial resistance. 1 The extensive utilization of antibiotics in clinical practice has been determined to be a leading factor for the emergence of antibiotic resistance. 2 Inefficient therapy due to resistance is associated with increasing human suffering, loss of productivity, and often ending with death. Antibiotics misuse and a high level of antibiotics resistance (AMR) are observed particularly in developing countries. Kosovo, a country located in Southeast Europe with a population of 1.7 million inhabitants, is facing some challenges regarding antimicrobial resistance such as limited financial and human resources, over-the-counter sale of antibiotics, and scarcity of clinical guidelines authorized by the Ministry of Health. 3 Kosovo is two to five times higher for the majority resistance of bacteria and corresponding antibiotics groups compared with the means in European countries. 4

To address the challenge of antimicrobial resistance, the Ministry of Health in Kosovo adopted (2011–2015) the first National Strategy and Action Plan to Combat Antimicrobial Resistance, which resulted in the surveillance of general antibiotics consumption. 3 Despite the high level of knowledge of health care professionals and great awareness of antimicrobial resistance, prescribing and dispensing practices were influenced by challenges of access to information on resistance patterns, next line antibiotics, diagnostics, and patient records. 5 Second National Action Plan for Antimicrobial Resistance was adopted (2019–2021) by the Ministry of Health regarding reports from various researches dealing with resistance occurrence, 4 6 which was also aimed at improving the monitoring capacity and optimization of antibiotics' use. The latest World Health Organization (WHO) data in 2018 showed that Kosovo made progress in appointing an AMR focal point by the Ministry of Health as well as an intersectoral coordinating mechanism to control AMR. 7 Also, in 2019, country representatives in a regional workshop held in Belgrade, Serbia reported a significant decrease in antibiotic consumption (25%). 8 However, a fully functional AMR reference laboratory with a quality assessment system in place is still missing.

Dental practitioners are frequently prescribing antibiotics for their patients as outpatient care. Among the reasons for the misuse of antibiotics in dentistry are the dentists' desire to avoid clinical complications, the fear to lose patients, and perceived patient pressure. 9 Recent research studies advocated that dental practitioners should prescribe antibiotics/antimicrobials only for acute orodental conditions, where drainage or debridement is impossible, where there is a local spread of infection, or where systemic upset has occurred, 10 but not for mere inflammation. 11 12 Additionally, prophylactic use of antimicrobials 13 in dental treatment is strictly recommended with special guidelines for suspected systemic infective conditions, such as infective endocarditis 14 15 and severely immunocompromised patients. The rational and effective prescription of antimicrobial is imperative in dental practice, and it is necessary to implement an antimicrobial prescription monitoring system and an antibiotic administration program. In addition, quality indicators of antibiotic prescription are needed to be continuously updated according to the standards recommended by WHO.

Therefore, the patterns of antibiotics prescription of dentists in Republic of Kosovo major dental clinics are investigated by this research regarding the extent to which and for what reasons they prescribe antibiotics. The purpose of the present study is to investigate patterns of antibiotics prescription of dentists in Kosovo's major dental clinics.

## Materials and Methods

This study consisted of data collection regarding antibiotics use during 2015 to 2019 at major dental clinics in the Republic of Kosovo. Data collection based on Point Prevalence Survey (PPS) study was performed in 10 regional Dental Clinics and a Tertiary Health Center. Data collection was performed by the author of this study in collaboration with the clinics' experienced staff after ethical approval in May 2019 by the management board of the hospitals and University Clinical Service of Kosovo by app. no. 830/2. Ethical approval for collaboration in research was granted by the Ethic committee of the School of Dental Medicine University in Zagreb 05-PA-30-VII—5/2019.

### Data Collection

Patients' forms were designed based on PPS methodology and contain information on identification of the patients' number, year, age, gender, diagnosis, generic name of the drug used, dosage, the single-dose administered in units, and duration of the treatment. The patient form is developed by the European Antibiotics Use Surveillance 16 and Antibiotics Resistance and Description of European Children. 17

Data were gathered and analyzed for the University Clinical Dentistry Center of Kosovo in Prishtina; 18 Family Health Care Centers in Prishtina; and 10 Main Centers of Family Medicine in Vushtrri, Prizren, Mitrovice, Obiliq, Ferizaj, Hani i Elezit, Gjilan, Gjakovë, Fushë Kosovë, and Pejë. Inclusion criteria for data collection were completed antibiotic treatment for a relevant disease in dental medicine in mentioned dental clinics during the period from 2015 to 2019. Emergency patients which had received immediate (short) therapy and were directed to the dentist for further treatment were excluded because of the lack of complete documentation.

### Antibiotic Utilization Data

For analysis of the antibiotic utilization data, the defined daily dose (DDD) and the defined daily dose per 1,000 inhabitants per day (DID) formula codes were used to calculate a standardized measure of medicine consumption at the national level. DDD is defined as the assumed average maintenance dose per day for a drug used for its main indication in adults, and this standard value for each drug was obtained from the World Health Organization Collaborating Centre for Drug Statistics Methodology website ( https://www.whocc.no/atc_ddd_index/ ), and it has become a major assessment tool in the pharmacoepidemiologic studies since it provides a fixed unit of measurement independent of dosage form, package size, or price.

### Statistical Analysis

Quantitative data are analyzed by statistical analysis where data are transformed into numbers, percentages, tables, and diagrammatic representations. Data were entered in MS Excel database according to the year, number of health care centers, most used antibiotics, most common diagnosis, and number of patients for both of them. The empirical analysis as the basis of this study is used to arrive at the most complete conclusions.

## Results

On average, there were 181 antibiotic prescriptions issued per dentist/annually, with an average number of 47% of antibiotic prescriptions per patient who used some kind of service in observed dental clinics. For the analyzed period, the average antibiotic utilization by each dentist was 6.5 DDD/1,000 patients/year, with fluctuations between the years 2015 and 2019. The number of dentists in Kosovo in 2019 was 1,500 and the number of dentists increased by 22% during the observed period. The number of patients that used dental services was on average 1,265 annually with minimal fluctuations. According to a statistical agency report on population estimation, Kosovo had 1,771,604 inhabitants in 2015 and the number increased by 0.59% in the next 5 years. 18 19

All data regarding antibiotics prescription in dentistry in Kosovo are presented in Table 1 according to year, number of dental health care centers; number of patients in total, most used antibiotics, number of patients who received them, and most common diagnosis and number of patients who were diagnosed. There was a decrease in utilization observed from 2017 to 2019 in overall antibiotics prescriptions in each of the cities. Results from antibiotics use during the period from 2015 and 2019 are presented in Table 2 for each city, with Gjilan having a significant descending trend of antibiotics use and Peja having a significant rising trend. More information on the most common diagnosis for each dental center in Kosovo in the years 2015 to 2019 is presented in Supplementary Tables S1 – S5 (available in the online version only).

**Table 1:** Antibiotics' prescriptions in dentistry in Kosovo during years 2015 to 2019

Year	No. of health care centers	No. of patients (total)	Most used antibiotics	No. of patients	DDDs	DDD/1,000 patients/year	Most common diagnosis	No. of patients
2015	11	1,115	Cefazolin	235	1,175	2.88	KO4.7: Periapical abscess without sinus	139
			Amoxicillin	177	885	2.17	KO4.5: Chronic apical periodontitis	87
			Cefalexin	139	695	1.70	KO4.0: pulpitis	55
2016	16	1,373	Amoxicillin	294	1,470	2.92	KO4.7: Periapical abscess without sinus	293
			Cefazolin	190	950	1.89	KO4.1: Necrosis of the pulp	70
			Cefalexin	120	600	1.19	KO4.0: Pulpitis	61
2017	17	1,641	Amoxicillin	351	1,755	2.91	KO4.7: Periapical abscess without sinus	443
			Cefazolin	255	1,275	2.11	KO4.1: Necrosis of the pulp	122
			Amoxiclav	216	1,080	1.79	KO4.5: Chronic apical periodontitis	76
2018	17	1,336	Amoxiclav	216	1,080	2.16	KO4.7: Periapical abscess without sinus	349
			Cefazolin	207	1,035	2.07	KO4.1: Necrosis of the pulp	135
			Amoxicillin	74	370	0.74	KO4.5: Chronic apical periodontitis	67
2019	17	862	Amoxiclav	215	1,075	3.41	KO4.7: Periapical abscess without sinus	210
			Amoxicillin	164	820	2.59	KO4.1: Necrosis of the pulp	109
			Cefazolin	126	630	1.99	KO4.5: Periapical abscess without sinus	32

Abbreviation: DDD, defined daily dose.

**Table 2:** Most prescribed antibiotics by the region cities and each dental center in Kosovo in 2015 and 2019

Place	Year	Most prescribed antibiotic 1	No. of rx.	Most prescribed antibiotic 2	No. of rx.	Most prescribed antibiotic 3	No. of rx.
UCDCK in Prishtinë	2015	Amoxicillin + clavulanic acid	6	Amoxicillin	1	No data	/
	2019	Amoxicillin + clavulanic acid	63	Metronidazole	14	No data	/
MCFM in Vushtrri	2015	Amoxicillin	68	Amoxicillin + clavulanic acid	19	Ceftriaxone	11
	2019	Amoxicillin	26	Amoxicillin + clavulanic acid	5	No data	/
MCFM in Prizren	2015	Amoxicillin	27	No data		No data	/
	2019	Amoxicillin	19	No data		No data	/
MCFM for maxille	2015	Cefazolin	235	Ampicillin	63	Cefalexin	54
	2019	Cefazolin	126	Amoxicillin + clavulanic acid	30	Metronidazole	21
MCFM in Mitrovice	2015	Cefalexin	44	Ceftriaxone	16	Amoxicillin	12
	2019	Cefalexin	26	Amoxicillin	16	Amoxicillin + clavulanic acid	13
MCFM in Ferizaj	2015	Amoxicillin	31	No data		No data	/
	2019	Amoxicillin	19	No data		No data	/
MCFM in Gjilan	2015	Amoxicillin	79	Penicillin G	45	Cefalexin	41
	2019	Amoxicillin	27	Penicillin G	5	Amoxicillin + clavulanic acid	8
MCFM in Fushë Kosovë	2015	Amoxicillin	22	Ceftriaxone	15	Amoxicillin + clavulanic acid	11
	2019	Amoxicillin + clavulanic acid	26	Ceftriaxone	19	Amoxicillin	16
MCFM in Pejë	2015	Amoxicillin + clavulanic acid	19	Amoxicillin	29	No data	/
	2019	Amoxicillin	71	Amoxicillin + clavulanic acid	27	No data	/

Abbreviations: MFMC, Main Family Medicine Center; UCDCK, University Clinical Dentistry Centre of Kosovo.

Among antibacterials, amoxicillin was the most prescribed, averaging 36% of all antibiotic prescriptions, which followed by cefazolin (34%), amoxicillin with clavulanic (26%), and cefalexin (9%). A trend of high increase in the use of amoxicillin with clavulanic acid was observed from 1.79 DDD/1,000 patients/year to 3.41 DDD/patient/year over 5 years.

## Discussion

The aims of this study were to perform a descriptive analysis and quality assurance of antibiotic prescriptions at major dental clinics in the Republic of Kosovo during 2015 to 2019. The most prescribed antibiotics by results of this study in secondary/tertiary dental health care in Kosovo were amoxicillin (16.7%), cefazolin (16%), amoxiclav (10.2%), and cefalexin (4.09%). Amoxicillin, as most prescribed, was the first choice in dental infection treatment as is recommended by guidelines. 10 11 12 However, authors must warn of the observed trend of a dramatic increase in amoxicillin with clavulanic acid prescription which is occurring in the past 2 years, increasing from 1.16 DID to 3.86 DID. If this trend is to be continued, then the wider spectrum antibiotic will prevail as the first choice leading to new problems regarding microbial resistance. A similar trend in increasing usage of amoxicillin with clavulanic acid was observed in the latest research from Croatia and Turkey. In Croatia, the most used antibiotics are amoxicillin with clavulanic acid, amoxicillin, and clindamycin, respectively, according to studies by national health insurance data and cross-sectional self-reported study. 20 21 Amoxicillin is part of the penicillin group of antibiotics, but is effective against a broader range of organisms and recommendations for antibiotic treatments in dentistry are to start with a narrower one (amoxicillin), so the broader one could be effective when needed. Observing these differences in results, with a great increase in amoxicillin with clavulanic acid, we may suggest that a shift has occurred in the first-choice antibiotic. It could be because of the differences in diagnosis and indications for antibiotic treatment. According to our study, the highest number of patients in health care dental clinics in 2015 to 2019 received antibiotics for maxilla-related health conditions, and the lowest number of them for oncologic ones. But further studies need to be done to confirm this observation.

The data in our research are based on the data of patients from secondary and tertiary health care facilities, with complex diagnoses, who are most often referred for treatment from primary health care institutions. Kosovo still has not implemented systemic monitoring of antibiotics utilization in primary dental health care, which presents a big problem in rationalizing antibiotics utilization in dental practice. The trends in antibiotics utilization in primary dental practice were partially illuminated by the Hailiti et al 22 research, which required manually retrieving the data of each patient in 12 ordinations of primary dental practice. In primary dental offices where the study was conducted during March to June 2012, the most commonly prescribed antibiotic was amoxicillin with clavulanic acid (43.45%), which followed by metronidazole (2.31 DDD) and amoxicillin (1.25 DDD). 22 By comparing results, it is obvious that the rationalization in antibiotics utilization is more present in secondary/tertiary facilities than in primary health care. This can be primarily be seen from lower DDD values but secondarily from the first-choice antibiotic, amoxicillin against amoxicillin with clavulanic acid. The higher level of rationalization in tertiary care can possibly be explained by research, where dentists working in a university/hospital setting are more secure in making decisions regarding treatments and following protocols and accurate guidelines. 23

Regarding the dosage and duration of antibiotic therapy, the most prescribed antibiotic was 500 mg of amoxicillin administered 3 × 1 for 5 days. The dosage and number of days are prescribed according to the clinical characteristics of patients. For cefazolin, the prescriptions were 3 g per os for 5 days, for cefalexin 1.5 g per os for 5 days, and for amoxiclav 1.5 g per os for 5 days as well. A similarity with the Croatian study was in the most common indication for antibiotics prescription, in Kosovo for KO4.7 (periapical abscess) without sinus and in Croatia for periapical or periodontal abscess, 21 a fact which is similar also with findings of Palmer et al. 24 This pattern of antibiotic prescribing is found to be accurate with the actual recommendations. But this result has to be confirmed with a further study determining prescription patterns for primary private dental health care, which are not part of clinics.

Although on a national level, a positive trend in rational prescription is observed, when analyzing each city separately, the case is different for each one. Regarding the scale of the rational use of antibiotics, in the case of Vushtrri, antibiotics were frequently prescribed for KO4.4 (acute apical periodontitis of pulpal origin), even though the clinical relevance of bacteria being present in the tissues is still not clearly defined in these infections. 25 Regarding antibiotics prescriptions for KO4.1 (necrosis of the pulp) in FHCC V “Dardani,” Prishtinë, these drugs cannot achieve an adequate therapeutic concentration within the necrotic pulp 26 as well as are not needed in periapical infections because of meticulous endodontic technique use. 27 In the Family Health Care Center IX “Vneshta,” Prishtinë, antibiotics were frequently prescribed for KO4.2 (pulp degeneration) and some other studies have mentioned overprescription of these drugs in this case (irreversible pulpitis). 28 In the Family Health Care Center 1, Prishtinë, the most common dental health condition for antibiotics prescription was also KO3.6 (deposits on teeth), which requires plaque removal and hygiene measures. In the Main Center of Family Medicine in Obiliq, the second most common dental condition for antibiotics' prescription was ZO1.2 (dental examination) even though antibiotics are not needed for simple dental examinations without defining a proper diagnosis. In the Main Center of Family Medicine in Hani i Elezit, KO4.0 (pulpitis) was the most frequent condition for antibiotics prescription by dentists, even though pulpitis is a clinical case, on which antibiotics are not needed. 29 The treatment of pulpitis, as well as periapical periodontitis, requires only operative measures. 30 Also, in Gjilan, KO4.5 was the most common dental condition treated with antibiotics, even though the routine use of systemic antibiotics in the treatment of chronic periodontitis is not justified in normal healthy patients because of the related risks. 31 In these cases, the removal of the calculus and infected tissue by scaling and root planing procedure with irrigation removes the infectious foci and resolves the inflammation. 25

There is currently a culture of antibiotic overuse in Kosovo, including attitudes and behaviors, and hence also experiences of patients, pharmacists, and physicians, which is possibly underlying the high consumption of antibiotics in the country. 5 32 However, a level of awareness exists of the current problematic situation among practitioners and policy makers. 5 In a study conducted in 2011 in Kosovo, antimicrobial use in 2011 was 26.3% DDD per 1,000 inhabitants per day and 56.8% of inpatients were using at least one antibiotic with ceftriaxone, as the most prescribed antibiotic, 33 while data on antibiotic usage in dentistry to the date are unavailable. Previous studies have shown a high prescription rate of antibiotics in dental care in Kosovo. 34 35 Most of the antibiotics prescriptions are empirical and use broad-spectrum antibiotics compared with narrow-spectrum ones. Although there are recommendations about antibiotic therapy in dentistry, there are no surveillance measures or research done to corroborate if dentists follow national policy measures and recommendations.

The limitation of the study is the lack of information on the prescription pattern from general and private dentistry practice, which would provide better insight into general antibiotic utilization from overall dentistry practice. The problem seems to be structural in nature; since the data collection method differs for each region and type of practice, many of them still uncategorized.

Based on the above findings, although there is an observed decreasing trend of antibiotic use in Kosovo on a national level in the years 2015 to 2019, many separate and smaller cities and places need improvement in antibiotic utilization. The decreasing trend is a positive result of the national antibiotic awareness program and should be continued and adjusted for observed differences. In the end, it can be stated, in light of the global dental health care standards, the irrational use of antibiotics in Kosovo remains a problem, which needs to be monitored and further addressed.

## Conclusion

The results demonstrated that the patterns of antibiotics prescriptions by dental practitioners in Kosovo during the years 2015 to 2019 have an improving trend because fewer antibiotics have been used in secondary/tertiary dental clinics. Amoxicillin was the first-choice antibiotic as recommended, but co-amoxiclav utilization has shown a rapidly increasing trend which is a sign for alert and closer surveillance of antibiotic utilization in dentistry. Compared with the global health care standards, the irrational use of antibiotics in dental health care clinics in Kosovo still exist, and this issue should be further addressed by respective actors. There has been great inconsistency in prescribing patterns for different regions of Kosovo, which needs to be addressed in further investigations. Rational use of antibiotics in dental health care is imperative not only for dentists but also for pharmacists and patients. Kosovo and other Western Balkan countries need to review and/or fully align their national legal framework to enable effective implementation of EU legislation relating to AMR and EU standards; to set specific and measurable targets as a basis for national strategies and action plans and to have regular information updates on AMR and antimicrobial consumption available for proper monitoring against targets.
