# Supplementary material for: Administration of Systemic Antibiotics for Dental Treatment in Kosovo Major Dental Clinics: A National Survey
Source: Eur J Dent. 2022 Jan 11;16(2):430–6. doi: 10.1055/s-0041-1735931 (PMC9339925; doi:10.1055/s-0041-1735931)
Supplement: Supplementary file 1 — Supplementary Material [file 10-1055-s-0041-1735931-s2151584.pdf]

Supplementary Table S1 Most common diagnosis for each dental center in Kosovo in 2015

Place	Year	Most common diagnosis 1	No. of Rx	Most common diagnosis 2	No. of Rx	Most common diagnosis 3	No. of Rx
UCDCK in Prishtinë	2015	KO4.7	3	KO4.5	2	KO8.3	1
MCFM in Vushtrri	2015	KO4.7	53	KO4.1	37	KO8.3	7
MCFM in Prizren	2015	KO4.02	19	KO8.3	6	KO4.7	4
MCFM for maxille	2015						
MCFM in Mitrovice	2015	KO4.7	33	KO4.5	20	KO5.0	9
MCFM in Ferizaj	2015	KO4.02	19	KO8.3	5	KO4.7	4
MCFM in Hani i Elezit	2015	KO4.0	55	K12.2	13	KO4.1	8
MCFM in Gjilan	2015	KO4.5	87	KO4.6	24	KO8.1	13
MCFM in Fushë Kosovë	2015	KO4.7	42	KO8.1	9	No data	
MCFM in Pejë	2015	No data		No data		No data	
MCFM for oncological issues	2015	Ca. planocelulare	24	Ca. labialis inf	8	No data	

Abbreviations: MFMC, Main Family Medicine Center; UCDCK, University Clinical Dentistry Centre of Kosovo.

Supplementary Table S2 Most common diagnosis for each dental center in Kosovo in 2016

Place	Year	Most common diagnosis 1	No. of Rx	Most common diagnosis 2	No. of Rx	Most common diagnosis 3	No. of Rx
UCDCK in Prishtinë	2016	KO4.7	24	KO5.22	15	KO8.3	12
MCFM in Vushtrri	2016	KO4.4	35	KO4.7	25	KO4.1	14
FHCC IX “Vneshta” in Prishtinë	2016	KO4.1	18	No data		No data	
FHCC “Velani” in Prishtinë	2016	KO4.1	8	No data		No data	
FHCC “Bregu i Diellit” in Prishtinë	2016	KO4.1	22	No data		No data	
FHCC I in Prishtinë	2016	KO3.6	19	KO4.2	11		
MCFM in Prizren	2016	KO4.7	17	KO4.0	7	KO4.02	6
MCFM for maxille	2016						
MCFM in Mitrovice	2016	KO4.7	47	KO4.5	6	K10.3	4
MCFM in Obiliq	2016	KO4.7	50	KO8.3	7		
MCFM in Ferizaj	2016	KO4.7	22	KO4.0	13	KO8.3	6
MCFM in Hani i Elezit	2016	KO4.0	41	K12.2	19	KO4.1	8
MCFM in Gjilan	2016	KO4.5	71	KO4.7	26	KO8.3	22
MCFM in Fushë Kosovë	2016	KO4.7	82	K10.3	7		
MCFM in Pejë	2016						
MCFM for oncological issues	2016	Ca. planocelulare	15	Ca. laboratory inf.	5		

Abbreviations: MFMC, Main Family Medicine Center; UCDCK, University Clinical Dentistry Centre of Kosovo.

 © 2022. The Author(s).

Supplementary Table S3 Most common diagnosis for each dental center in Kosovo in 2017

Place	Year	Most common diagnosis 1	No. of Rx	Most common diagnosis 2	No. of Rx	Most common diagnosis 3	No. of Rx
UCDCK in Prishtinë	2017	KO4.7	40	KO5.22	16	KO4.5	10
MCFM in Vushtrri	2017	KO4.7	40	KO4.4	17	KO4.1	13
FHCC IX "Vneshta" in Prishtinë	2017	KO4.1	22	KO4.0	2	KO6.9	1
FHCC "Velani" in Prishtinë	2017	KO4.1	14	KO4.0	2		
FHCC "Bregu i Diellit" in Prishtinë	2017	KO4.1	33				
FHCC I in Prishtinë	2017	KO3.6	26	KO4.2	7		
MCFM in Prizren	2017	KO4.7	57	KO10.3	11	KO8.3	4
MCFM for maxille	2017						
MCFM in Mitrovice	2017	KO4.7	56	KO4.5	4		
MCFM in Obiliq	2017	KO4.7	85	ZO1.2	6		
MCFM in Ferizaj	2017	KO4.7	57	KO8.3	15		
MCFM in Hani i Elezit	2017	KO4.0	16	KO4.1	7	K12.2	4
MCFM in Gjilan	2017	KO4.5	62	KO4.0	11	KO8.3	7
MCFM in Gjakovë	2017	KO4.1	33	KO4.4	14	KO4.6	11
MCFM in Fushë Kosovë	2017	KO4.7	108	K10.3	6		
MCFM in Pejë	2017						
MCFM for oncological issues	2017	Ca. planocelulare	5	Ca. laboratory inf.	4		

Abbreviations: MFMC, Main Family Medicine Center; UCDCK, University Clinical Dentistry Centre of Kosovo.

Supplementary Table S4 Most common diagnosis for each dental center in Kosovo in 2018

Place	Year	Most common diagnosis 1	No. of Rx	Most common diagnosis 2	No. of Rx	Most common diagnosis 3	No. of Rx
UCDCK in Prishtinë	2018	KO4.7	22	KO5.22	12	KO8.3	7
MCFM in Vushtrri	2018	KO4.7	48	KO4.1	18	KO8.3	5
FHCC V "Dardani" in Prishtinë	2018	KO4.1	10	KO4.7	7	KO4.8	1
FHCC IX "Vneshta" in Prishtinë	2018	KO4.1	13	KO4.2	10	KO4.7	7
FHCC "Velani" in Prishtinë	2018	KO4.1	10	KO4.6	2		
FHCC "Bregu i Diellit" in Prishtinë	2018	KO4.1	25				
FHCC I in Prishtinë	2018	KO4.1	16	KO3.6	14	KO4.2	10
MCFM in Prizren	2018	KO4.7	22	KO4.2	3	KO4.1	3
MCFM for maxille	2018						
MCFM in Mitrovice	2018	KO4.7	95	KO4.5	5	KO8.3	4
MCFM in Obiliq	2018	KO4.7	9	ZO1.2	3		
MCFM in Ferizaj	2018	KO4.7	24	KO4.1	4		
MCFM in Gjilan	2018	KO4.5	62	KO4.0	11	KO8.3	7
MCFM in Gjakovë	2018	KO4.1	36	KO4.4	23	KO4.0	4
MCFM in Fushë Kosovë	2018	KO4.7	122	K10.3	7		
MCFM in Pejë	2018						
MCFM for oncological issues	2018	Ca. planocelulare	9	Sarcoma ewing	5		

Abbreviations: FHCC, Federal Health Care Center; MFMC, Main Family Medicine Center; UCDCK, University Clinical Dentistry Centre of Kosovo.

 © 2022. The Author(s).

Supplementary Table S5 Most common diagnosis for each dental center in Kosovo in 2019

Place	Year	Most common diagnosis 1	No. of Rx	Most common diagnosis 2	No. of Rx	Most common diagnosis 3	No. of Rx
University Clinical Dentistry Center of Kosovo in Prishtina	2019	KO4.7	29	KO4.5	14	KO8.3	12
The Main Center of Family Medicine in Vushtrri	2019	KO4.7	26	KO2.1	4	KO4.1	3
Family Health Care Center V “Dardani” in Prishtinë	2019	KO4.1	36	KO4.7	15	KO4.5	1
Family Health Care Center IX “Vneshta” in Prishtinë	2019	KO4.2	12	KO4.1	9	KO8.3	5
Family Health Care Center “Velani” in Prishtinë	2019	KO4.1	4	KO4.0	1		
Family Health Care Center “Bregu i Diellit” in Prishtinë	2019	KO4.1	14				
Family Health Care Center I in Prishtinë	2019	KO3.6	7	KO4.2	3		
The Main Center of Family Medicine in Prizren	2019	KO4.7	15	KO10.3	2		
The Main Center of Family Medicine for maxille	2019	Cefalexin	17				
The Main Center of Family Medicine in Mitrovice	2019	KO4.7	46	KO4.5	6	K10.3	4
The Main Center of Family Medicine in Obiliq	2019	KO4.7	4	ZO1.2	1		
The Main Center of Family Medicine in Ferizaj	2019	KO4.7	11	KO4.2	3	KO4.1	2
The Main Center of Family Medicine in Gjilan	2019	KO4.5	11	KO4.6	10	KO8.3	6
The Main Center of Family Medicine in Gjakovë	2019	KO4.1	43	KO4.4	3		
The Main Center of Family Medicine in Fushë Kosovë	2019	KO4.7	64	K10.3	5		
The Main Center of Family Medicine in Pejë	2019						
The Main Center of Family Medicine regarding oncological issues	2019	Ca. planocelulare	3	Sarcoma ewing	3		

 © 2022. The Author(s).
